# Combined interactions of amino acids and organic acids in heavy metal binding in plants

**DOI:** 10.1080/15592324.2022.2064072

**Published:** 2022-05-02

**Authors:** Ayhan Kocaman

**Affiliations:** Engineering Faculty, Environmental Engineering Department, Karabük University, Karabük, Turkey

**Keywords:** Heavy metal, organic acids, amino acids, proline, phytoremediation, bioaccumulation factors, hyperaccumulators

## Abstract

This research focused on the different approaches to the transport and internal chelation of metals with amino acids and organic acids in plants. Therefore, in the first phase, the plants studied were identified the characteristics of the bioaccumulation factors. *Steria pumila, Echium angustifolium, Typha angustifolia, Sisymbrium austriacum* were identified as hyperaccumulators (Cd, Ni), accumulators (Pb, Sn, and Se), excluders (Cr, Hg). On the other hand, the *Sisymbrium austriacum* only showed the characteristic of the accumulator for Cr. In the second phase, the combined effects of amino acids and organic acids on the chelation of heavy metals in plants were tested by a multi-linear regression model. Related to our hypothesis, Amino acids; Gly and Leu (Cd), Trp and Ile (Pb), Asp, Ser, and Leu (Cr), Ser (Hg), Trp and Glu (Ni), Asp, Thr, and Gly (Sn), Asn and Leu (Se), Organic acids; Malonic and Malic acid (Cd), Malonic acid (Pb), Oxalic and Malic acid (Cr), Oxalic, Succinic, Citric and Butyric acid (Hg), Malonic and Malic acid (Ni), Malonic, Maleic, and Malic acid (Sn), Malonic and Citric acid (Se) were concluded that had combined effect for heavy metal’s phytochelation ability into plants.

## Introduction

All plant species that are tolerant or sensitive to the stress of heavy metals have a basic defensive system that acts against the stress of heavy metals. Advances in biological studies and new approaches have begun to understand some of the complex strategies plants use at the cellular and molecular levels to fight against metal stress. The multifunctionality and versatile molecular properties of phytochelates (PCs) and metallothioneins (MTs) are gaining increasing attention because of the detoxification of heavy metals and the maintenance of cellular ionic equilibrium. PCs and MTs have the potential to interact directly or indirectly with the plant’s antioxidant defense system. It also contribute to the transport and distribution of surplus ionized metals between roots and shoots, depending on time or tissue^1^

Metal hyperaccumulation is a physiological process through which plants store large amounts of metal ions in aboveground tissues without being exposed to toxic effects. For this, the metal ions should be moved from the soil, absorbed by the roots, transmitted to the xylem for delivering them to the aboveground tissues.^2^ Plant resistance against heavy metals (HM) can be categorized in two: plants either excluder or show toleration for high internal metal concentration. The former is achieved through extracellular precipitation, decreased metal concentration intake through biosorption, or enriched flow. Plants achieve HM tolerance through intracellular chelation of HMs by synthesis of organic acids (OA), amino acids (AA), heavy metal-binding ligands, or glutathione. Antioxidant protection systems and glyoxalase neutralize the harmful effects of Reactive oxygen species (ROS). When methylglyoxal is upregulated,^3^ the metabolic products get concentrated in plant vacuoles.^4^ Various root fluid substances, such as OAs and AAs, act as HM ligands to form stable HM complexes in the rhizosphere.^5^ Considering the mechanisms behind OA-assisted metal chelation, hyperaccumulator plants stabilize HMs in leaf vacuoles using vacuole transporters, while HMs photostabilizing plants retain HMs mostly in root vacuoles.^6^ Some metal-excluded plants have the ability to grow in heavy metal-contaminated soils and retain HMs in low concentrations above ground through phytostabilization^7^ Plants develop various strategies to exclude metals from their tissues through the function of their cell walls, plasma membranes, and mycorrhizae. These plants can protect metabolic sites such as shoots through mechanisms that restrict the movement of toxic metals outside of the rhizosphere or roots.^8^ OAs in cells inhibit HMs from remaining as free ions in the cytoplasm by complicating them and reducing their bioavailability to plants.^9^ Stress due to HMs results in the accumulation of certain types of AAs. Limiting their flow to plants by extrinsic binding of HMs to low-weighted molecular compounds excreted by roots comprises the first line of defense against HMs.^10^ Through this mechanism, strong bonds are formed by chelating metals with the carboxylate groups of citrates, malate, malonate, aconitate, oxalate, or tartrates, which act as electron donors. The roots of the plants also contain hydrated natural pectin polysaccharides, which are cultures that bond toxic metal ions.^11^

Due to their ability to bind metals, AAs and their by-products can be used to respond to metal toxicity. AAs facilitate chelation of HM ions in cells and xylem juice, thereby resulting in detoxification of HMs^12^ and increased resistance of plants to toxic metal ion levels^13^ However, there is still no clear relationship between the accumulation of metals and the production of these compounds.

Proline is a potential Osmo preservative that has been studied particularly in salinity- and drought-stressed crops. Its role in ROS sweeping and metal chelation in plants exposed to metals has been studied.^14^ Phytochelatin synthase (PCS) has been reported recently in plants exposed to high metal concentrations. High proline concentration plays a role in regulating the expression of melatonin-2, glutathione reductase, and glutathione synthetase genes.^15^ Although they are not directly related to the chelation of metals in plants, they regulate the water in proteins. By balancing the protein’s natural state, Osmo protectors help to preserve its structural properties and integrity.^16^ Generally, the proline concentration is the highest among the AA groups when plants are exposed to HMs.^17^ However, the concentration changes depending on the metals absorbed by the plants.^18^ The binding of metals to the constitutively expressed PCS enzyme is facilitated by Proline, as it increases the level of endogenous glutathione (GSH; γ-glutamyl-cysteinyl-glycine) and enables GSH to catalyze the conversion of reduced glutathione to PCS.^19^ Enzymatic protection is possible via proline-assisted reduction of the activity of free metal ions following the formation of a metal (Zn/Cd) pro-complex.^20^

This research was planned in two phases. The First phase: The heavy metal contents of four different types of plants collected on farmland and areas polluted by industrial waste were analyzed according to their heavy metal content. From the results of this analysis, four plant species were determined as hyperacumluter, accumulator, and excluder for various heavy metals. The second phase, examined the common effect of amino acids and organic acids on chelation/detoxification of heavy metals in plants with high tolerance to heavy metals.

## Material and methods

### Study area and preparation for analysis

This study was conducted in the Karabük province of Turkey. Four different species samples of plants *Steria pumila (SP), Echium angustifolium (EA), Typha angustifolia (TA), Sisymbrium Austriacum (SA)*, and soil samples were collected from a steel industry dump site (PA) representing an anthropogenic polluted area (N 41° 10’ 42,60146’ and E 32° 38ʹ42,07808’). As a reference, a few samples (NPA) were collected from the farming areas (N 41° 12’ 45,47’ and E 32° 40’ 38,09”). Each plants species collected from PA and NPA were mixed with each among them according to the area harvested. The plants were washed to remove any surface contaminants and sheared into three parts to separate the root, stem, and leaf. Then, the plant and soil samples were allowed to dry at 24°C for one day. It was determined heavy metals in plant samples, the procedures was followed, about 0.5 g plant samples (in three replicate) were supplemented with 10 ml nitric + perchloric acid mixture. Resultant samples were then wet-digested until having 1 ml sample. Following the wet digestion procedure, resultant solutions were diluted with distilled water and readings were performed in an ICP OES (Inductively Couple Plasma spectrophotometer) (Perkin-Elmer, Optima 4300 DV, ICP/OES, Shelton, CT 06484–4794, USA) for heavy metal contents.^21^

### Determination of amino acids

The determination of AAs is performed using column separation with phenyl isothiocyanate (PITC).^22^ Standard solutions were prepared. Samples of 10 µl were dissolved in a buffer solution of 100 µl and dried under high pressure for an hour. This was again dissolved in 100 μl of buffer solution by the addition of 5 μl of PITC. The PITC derivatives formed at room temperature 5 min after the reaction time^23^ were dissolved for a second time at high pressure. The PITC derivatives of AA formed in 0.05 M sodium acetate at pH 6.8. After dissolving it in a 9:1 (v/v) mixture of 0.1 M sodium acetate and 10% methanol in 40% acetonitrile,^24^ 10–20 µl was analyzed by HPLC.

### Determination of organic acids

For analytical measurement of OAs, 15.6 µML oxalic acid, 66.6 µML tartaric acid, 74.6 µML malic acid, 339 µM succinic acid, 96 µM malonic acid, 5.7 µML ascorbic acid, 1.7 µML maleic OAs containing a mixture of acid, 95.1 µML citric acid, and 1.7 µML fumaric acid were used.^24^ The standards were prepared, and each AA mixture was analyzed by HPLC for the highest points.

### Determination of the activity of antioxidant enzymes in plants (POD, CAT, SOD)

For the extraction of enzymes, 0.5 g of fresh plant leaves was taken, placed in a mortar, and ground until they turned into powder by adding liquid nitrogen. Then, 5 ml of cold homogenate buffer (0.1 M KH_2_PO_4_, pH 7.0, containing 1% PVP and 1 mm EDTA) was added, and the mixture was transferred to a tube and centrifuged at 15000xg and +4°C for 15 minutes. The supernatant obtained by centrifugation was used as a source for measuring the activity of antioxidant enzymes.^25,26^

Catalase (CAT) converts H_2_O_2_ into O_2_ and H_2_O in the activity measurement environment; its activity is monitored based on the resulting decrease in absorbance at 240 nm.^27^

The peroxidase (POD) activity was determined by monitoring the absorbance increase at 470 nm caused by the colored compound^28^ that is the product of the reaction in which fecal H_2_O_2_ is the substrate.^26^

Superoxide dismutase (SOD) activity was analyzed based on the spectrophotometric determination of the inhibition of blue nitro tetrazolium (NBT) photochemical reduction.^29,30^

### Translocation factor (TF) and bioaccumulation factor (BAF)

TF expresses the displacement of metals from roots to shoots by equation (1). It was calculated based on the methods suggested by^31^ and .

(1) $$TF=\frac{heavy\;metal\;concentration\;in\;shoot}{heavy\;metal\;concentration\;in\;root}$$

BAF explains the amount of metal absorption from the soil into the plant shoots by equation (2).

(2) $$BAF=\frac{heavy\;metal\;concentration\;in\;shoot}{heavy\;metal\;concentration\;in\;soil}$$

TF > 1 signifies that the plant effectively translocates trace metals from the roots to the shoots . BAF values are categorized as (excluder) 1< (accumulator) <10 (hyperaccumulator) .

## Statistical analysis

All data were examined by SPSS 22 software of ANOVA. Duncan’s test was carried out to identify significant differences in the means (p < .05). Based on the analytical results obtained, a multi-linear regression was conducted to model the relationship between amino acids and organic acids in heavy metal uptake in plants.

## Results

The TF and BAF provide very important information about the HM accumulation and absorption capacity of plants. Plants either store metals absorbed from the soil in their roots or transfer them to the stem.^32^ BAF values was calculated plants of *SP-EA-TA-SA* (Cd 12.33–15.29-21.12–28.69, Cr 0.54–0.71-0.97–1.34, Hg 0.19–0.21-0.23–0.16, Pb 6.27–7.41-6.98–6.42, Ni 25.72–29.29-27.82–23.36, Sn 1.02–1.15-1.10–1.01, Se 1.77–2.62-1.58–1.65. The TF and BAF values of the study samples with HM pollution (PA) were given in S1. In addationaly, the results of the analysis of pH, EC, OM %, and Calcif % of the nutrients and HM content of the soil samples taken from root regions were given in S2, S3, S4.

The nutrient content analysis of the leaf, stem, and root regions of the samples collected from PA showed statistically significant differences (p < .05) (S5). A significant decrease was observed in the nutrient content of the plant samples collected from the PA compared to NPA. This decrease obtained: N 54.35%, P 46.24%, K 40.05%, C 52.58%, Mg 22.77%, Na 53.36%, Zn 60.85%, Fe 16.01%, Mn 22.50%, Cu 25.72%, and B 36.16%. However, there was a 432.44% increase in the concentration of Cl.

**Figure 1.**
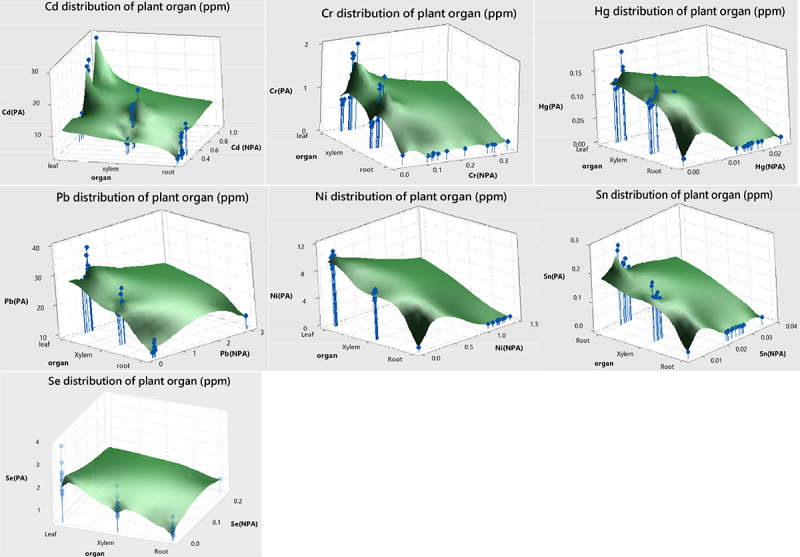
Distribution of heavy metal based on the plant organ.

HM content analysis in the leaf, stem, and root regions of samples collected from the PA showed statistically significant differences (p < .05). The regions with the highest concentration of HM were leaf > stem > root (Figure 1). However, there was no statistically significant difference observed in terms of the HM content in samples collected from the NPA. Moreover, the buildup of HMs in plant organs differed based on the plant species. (S6, Figure 1). Four plants content means of the PA samples showed higher concentrations of HMs than the NPA. These increases were determined as Cd 1778.30%, Cr 587.46%, Hg 855.23%, Pb 3371.31%, Ni 1040.47%, Sn 598.39%, Se and 21092.15%.

**Figure 2.**
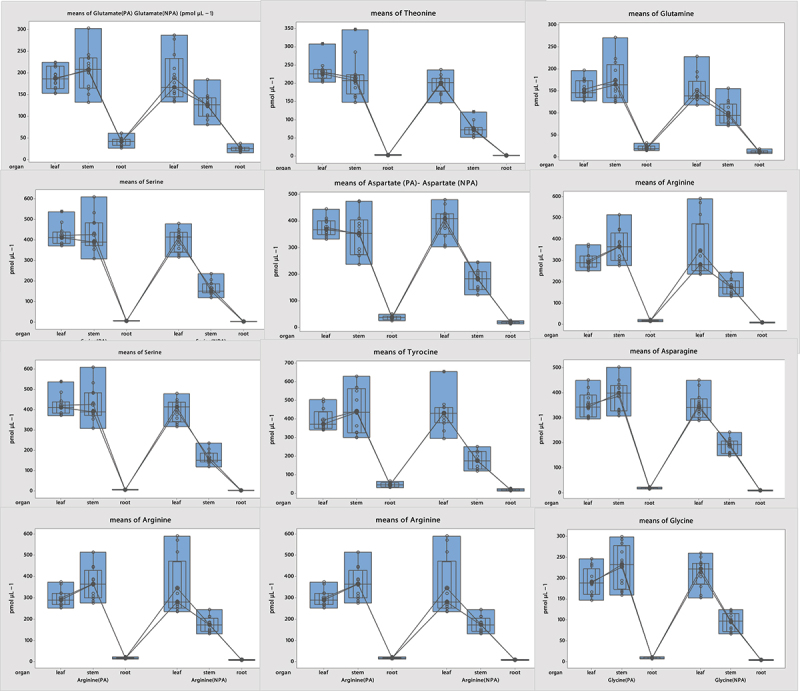
Continued.

AA metabolism plays an important role in the accumulation of osmolytes as part of the stress tolerance mechanism,^33^ regulation of intracellular pH, and detoxification of ROS, HMs, and xenobiotics.^34^ Statistically significant differences between plant organs in AA levels in PA samples (p < .05) were found. The highest AA content was usually on the stem ≥ leaf > root, respectively. But the highest content of AA in plants collected from the NPA was in the order of leaf > stem > root. In the plant samples from the PA, an increase in the AA content was seen in the stem portion. However, there were no statistical differences between the leaves and stems (S7, Figure 2). The AA content of the plant samples from the PA showed a significant increase compared to those from the NPA. These increases were calculated as Asp 28.12%, Glu 28.50%, Asn 39.22%, Ser 51.34%, Gln 32.14%, His 19.91%, Gly 37.02%, The 63.59%, Arg 29.02%, Ala 5.12%, Tyr 41.12%, Cys 24.00%, Val% 51.03, Met 43.27%, Trp 38.51%, Phe 28.22%, Ile 32.64%, Leu 41.07%, Lys 14.30%, Sarcosine 39.38%, Hyp 30.60% (Figure 2).
**Figure 2.**
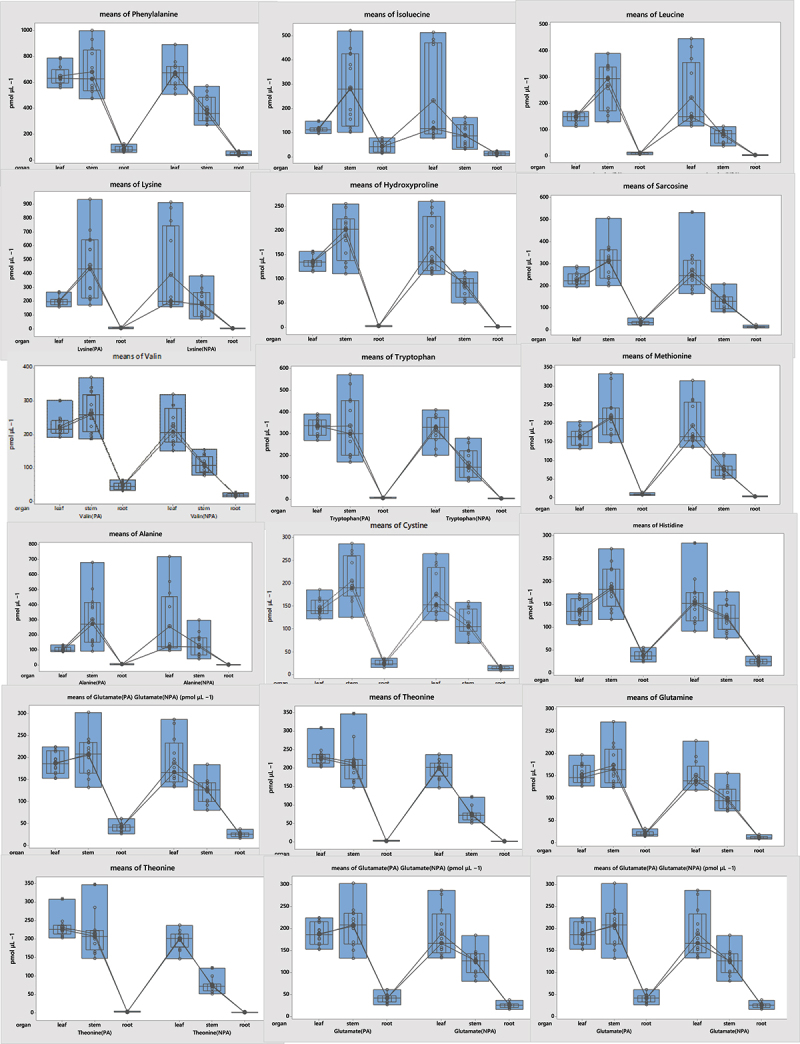
Amino acid content in plant organs.

**Figure 3.**
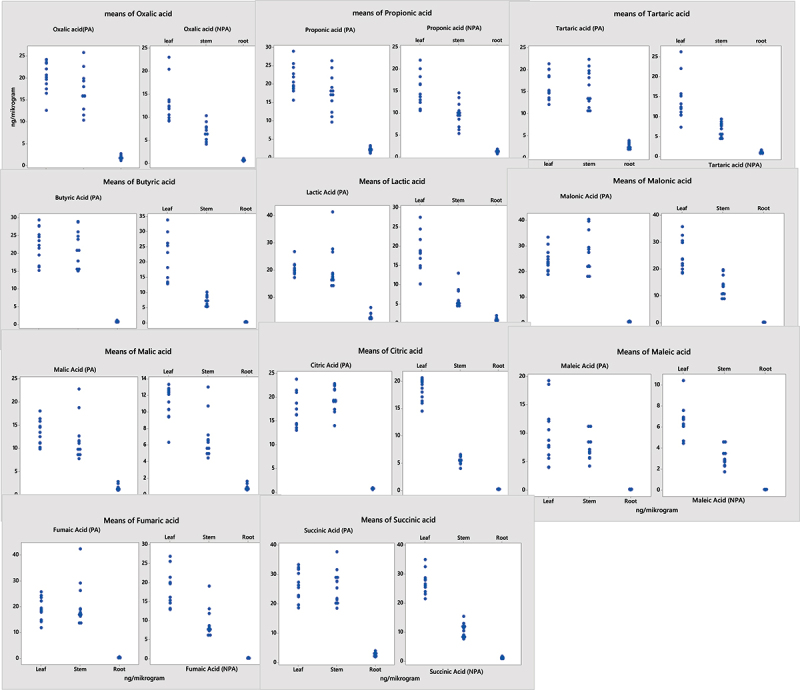
Organic acid content in plant organs from the NPA and PA.

Analysis of the OA content showed statistically significant differences among the organs (p < .05) (S8). The OA content in PA plant samples was found to be higher than in the NPA plant samples: Oxalic acid 91.02%, Propionic acid 60.70%, Tartaric acid 60.75%, Butyric acid 62.93%, Malonic acid 35.85%, Malic acid 44.64%, Lactic acid 72.43%, Citric acid 55.10%, Maleic acid 77.6%, Fumaric acid 44.77%, and Succinic acid 42.60% (Figure 3).

**Figure 4.**
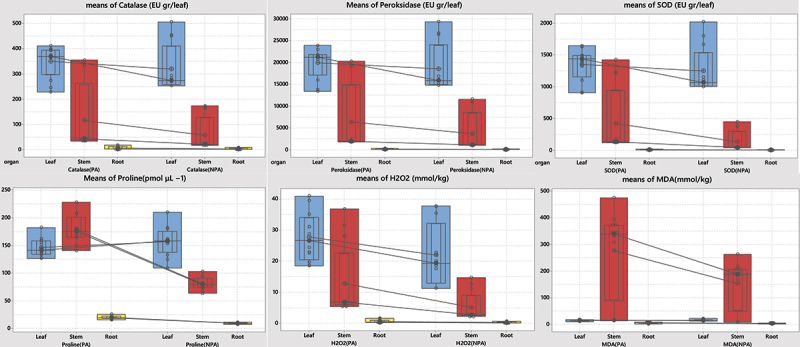
Distribution of antioxidants, H_2_O_2_, MDA, and proline content of plant samples from the NPA and PA.

Antioxidant and proline levels showed statistically significant differences among the plant regions (p < .05) (S9). It was found that the antioxidant, proline, MDA, and H_2_O_2_ content of plant samples from the PA was higher than that of the samples from the NPA. These increases was determineted as CAT 19.53%, POD 15.76%, SOD 22.25%, Proline 39.69%, H_2_O_2_ 33.61%, and MDA 42.54% (Figure 4, S10).

## Discussion

Plants either store metals absorbed from the soil in their roots or transfer them to the stem. Based on BAF, plants are classified as (excluder) 1< (accumulator) <10 (hyperaccumulator), respectively.^35^ According to the results of four plants of heavy metals content, Plants (*SP, EA, SA* and *TA*) were identified as Cd and Ni hyperaccumulator, Pb, Sn, and Se accumulator. They were also plants Cr and Hg excluder. On the other hand, the *SA* only showed the characteristic of the accumulator for Cr.

AAs are important metabolites with different functions, such as cell osmotic regulation, absorption of mineral nutrients, detoxification of HMs, and signaling.^17,36–38^ In this study, a linear, multivariate regression model was used to determine which AA in four different plant samples from PA were effective in the detoxification of different heavy metals in plants. Therefore, the best linear regression model was adjusted as a result of linear correlations of AAs for all plant species (Table 1). As a result of Cd amount of four plant species collected from PA in this study, there were differences in terms of the correlation between Cd uptake and AAs. For this reason, a linear multivariate regression model was used to see which AAs showed a higher linear correlation with the Cd content of plant samples. As a result of the adjusted of best linear model, the interaction of Gly, Thr, Tyr, Lys, and Leu in phytochelatin of the Cd is explained at a rate of 73.29% (p < .001) (Table 1). It was estimated that 1 unit increase in the amount of Gly and Leu would contribute to the 0.56 and 0.10 units chelation of Cd into plants. Whereas, in a study was reported on lettuce that Glu, Tyr, Lys, Asp play a role in the formation of a cadmium-containing PCS complex and protect it.^39^ Another study, Try, Lys, and His was observed accumulated more in all tissues of *Massai grass* exposed to Cd, and reported that these amino acids were probably implicated in Cd accumulation and detoxification in this process.^40^ Furthermore, a study showed that the AA contents in the root exudates from the rice line with high Cd accumulation, except for Tyr, which increased significantly as the Cd increased. Whereas, in the control group, only Met, Lys and His increased.^41^

**Table 1.** Multilinear regression model for heavy metal detoxification by amino acids.

HM	Model building		R-sq %	Final model Equation
Cd	X1:Gly X2:Thr X3:Tyr X4:Lys X5:Leu	73.29	=12.88 + 0.561 X1- 0.157 X2- 0.2103 X3- 0.0628 × 4 + 0.0959 X5- 0.001257 X2^2- 0.000346 X3^2 – 0.002544 X1*X2 + 0.002757 X2*X3 + 0.000445 X2*X4 – 0.000443 X3*X5	
Cr	X1:Asp X2:Glu X3:Asn X4:Ser X5:Leu	82.36	=0.299 + 0.01146 X1- 0.01035 X2- 0.0271 × 3 + 0.00096 X4 + 0.03371 X5- 0.000035 X1^2 + 0.000073 X1*X3- 0.000059 X1*X5 + 0.000045 X2*X4-0.000037 X4*X5	
Hg	X1:Tyr X2:Ser	92.26	= −0.00408 + 0.000212 X1 + 0.000379 X2 − 0.000001X1*X2	
Pb	X1:Asn X2:Val X3:Trp X4:Ile	90.32	=10.56–0.1077 × 1–0.0141 X2 + 0.2007 X3 + 0.0745 X4-0.000312 X3^2 + 0.000341 X1*X2 – 0.000324 X2*X4	
Ni	X1:Phe X2:Trp X3:Lys X4:Glu	96.87	=−1.143–0.00276 × 1 + 0.0457 X2 – 0.00838 × 3 + 0.03069 X4 + 0.000023 X1^2 – 0.000078 X1*X2	
Sn	X1: Lys X2:Asp X3:Thr X4:Gly	92.50	=−0.0047–0.000679 × 1 + 0.000434 X2 + 0.000699 X3 + 0.001985 X4 + 0.000001 X1^2-0.000003 X2*X4 – 0.000004 X3*X4	
Se	X1:Asn X2:Arg X3:Phe X4:Ile X5:Leu	77.50	== 1.320 + 0.00391 X1- 0.0120 X2- 0.00708 X3- 0.00351 × 4 + 0.0449 X5 + 0.000086 X2^2- 0.000022 X1*X4- 0.000179 X2*X5 + 0.000034 X3*X4	

The interaction of Asn, Val, Trp, Ile in phytochelatin of the Pb is explained at a rate of 90.32% (p < .001) (Table 1). It was estimated that 1 unit increase in the amount of Trp and Ile would contribute to the 0.20 and 0.08 units chelation of Pb into plants. A statistically significant increase in *Vetiver*, Asn, Pro, His, Trp, Val, Ile, Thr, Nicotinamide, and Met was observed under Pb stress.^42^ On the other hand, it was found that higher levels of Pb removed AAs from radish roots.^43^

The interaction of Asp, Glu, Asn, Ser, Leu in phytochelatin of the Cr is explained at a rate of 82.36% (p < .001) (Table 1). It was estimated that a 1 unit increase in the amount of Asp, Ser and Leu would contribute to the 0.011, 0.0009, and 0.033 units chelation of Cr into plants. The underlying events of defense signal transduction against Cr toxicity haven’t been partially elucidated. Glutamic acid, Ser, glutamine, β-alanine, Tyr, Cys, Met, and Ile^44^ content of the samples prepared from *the Juncea* seedlings grown in Cr solutions decreased in comparison with control seedlings.^45^ But their Lys and Pro contents increased compared to the control seedlings.^44^

High Hg intake in plants may cause adverse phytotoxic effects.^46^ Although largely retained in the roots, small amounts of accumulation can also occur in shoots by displacement of soluble forms or uptake of the gaseous form directly through the leaves.^47^ Relatively little is known about the molecular mode of action and defense responses of Hg stress. In our research, Tyr and Ser were found responsible for phytochelation of Hg with 92.26%. (p < .001); Notably, Ser alone acted as a phytochelatin at the rate of 92.17%. This regression effect demonstrated that Ser may be an important AA in Hg chelation. It was estimated that a 1 unit increase in the amount of Ser would contribute to the 0.0004 units chelation of Hg into plants. Many researchs of literature refer to Glu-Cys and Gly as phytochelatins of Hg. For this reason, studies on the role of serine in Hg uptake and chelation should be undertaken.^48^It was reported that inorganic Hg can primarily affect soluble compartments, while methyl Hg primarily affects insoluble compartments, and that levels of Phe, Thr, and Try increased in *Elodea nuttallii* shoots under stress.^49^

The interaction of Phe, Trp, Lys, Glu, in phytochelatin of the Ni is explained at a rate of 96.87% (p < .001) (Table 1). Ni concentrations of the plants, it was estimated that an increase of 1 unit in the amount of Trp and Glu would contribute to the chelation of 0.05 and 0.031 units of Ni in the plants. In the study by,^50^ *alyssum*, a Ni hyperaccumulator, was found to generate His proportional when exposed to nickel. Moreover, they found no uptake of nickel His in the non-Ni hyperaccumulator. In additionally, a study on wheat showed that, compared to control, the highest Ni uptake occurred in the presence of Gln. However, the use of both Gln and Gly significantly increased the root Ni accumulation, while His did not have a significant effect on the Ni accumulation in the roots.^51^

The interaction of Asp, Thr, Lys, Gly, in phytochelatin of the Sn was explained at a rate of 92.50% (p < .001) (Table 1). Sn concentrations of the plants was estimated that an increase of 1 unit in the amount of Asp, Thr and Gly would contribute to the chelation of 0.0004, 0.0007, and 0.002 units of Sn in the plants. In additional, Asn, Arg, Phe, Ile, leu only showed an effect on Se at the rate of 77.50% (p < .001) (Table 1). Se concentrations of the plants was estimated that an increase of 1 unit in the amount of Asn and Leu would contribute to the chelation of 0.004 and 0.05 units of Se in the plants. Some of studies showed, the application of Se foliar to *potato tubercles* has been shown to increase significantly, especially Asp, Glu, Tyr, Thr, and Phe, when compared to controls^52^. It has also been reported that Gly reduces the lethal effects of HMs by improving the resistance mechanisms of plants under stress.^53^

The metabolism of OAs is of fundamental importance on the cellular and the entire plant level. Over the past few years, more attention has been paid to the role of OAs in the modulation of environmental adaptation,^54^ including the contribution of OAs to the detoxification of HMs. The basis of this is the ability of OAs like citrate, malate, oxalate, malonate, citrate, and tartaric acids to form strong bonds with HM ions by chelation of metals, as well as their effect on the carboxyl groups^55^ which transport donor oxygen functions in metal ligands.

In our study, a statistically significant correlation was found between OA levels and HM concentrations in plants (p < .001). Therefore, a linear, multivariate regression model was tested to determine which OA’ amounts in four different plant samples from PA were effective in the detoxification of different heavy metals in plants. Thus, the best linear regression model was adjusted as a result of linear correlations of OAs for all plant species (Table 2).

**Table 2.** Multilinear regression model for heavy metal detoxification by organic acids.

HM	Model building	R-sq %	Final model Equation
Cd	X1:Oxalic acid X2:Malonic acid X3:Malic acid X4:Lactic acid X5:Citric acid	67.86	=14.87–2.040 X1+ 5.60 X2 + 2.03 X3-1.623 X4- 5.53 X5- 0.1086 X2^2- 0.1286 X3^2 + 0.1205 X1*X5 + 0.1583 X4*X5
Cr	X1:Oxalic acid X2:Propionic acid X3:Malic acid X4:Lactic acid X5:Maleic acid	71.58	=−0.011 + 0.0936 X1- 0.0319 ×2 + 0.197 X3- 0.0756 X4- 0.01491 X1*X3 + 0.00654 X2*X4
Hg	X1:Oxalic acid X2:Malonic acid X3:Succinic acid X4:Bütyric acid X5:Citric acid	98.72	=−0.00678 + 0.000924 X1- 0.00263 ×2 + 0.001110 X3 + 0.00985 X4 + 0.00278 X5 + 0.000413 X4^- 0.000417 X2*X4 + 0.000584 X2*X5 – 0.000905 X4*X5
Pb	X1:Oxalic acid X2:Malonic acid X3:Succinic acid X4: Malic acidX5: Citric acid	98.23	=16.54–0.461 ×1+ 4.406 X2- 0.584 X3- 1.224 X4- 3.747 ×5 + 0.0842 X1^2- 0.06360 X2^2- 0.1083 X4^2 + 0.0692 X5^2- 0.0746 X1*X2 + 0.1004 X3*X4 + 0.0635 X4*X5
Ni	X1:Oxalic acid X2:Malonic acid X3:Succinic acid X4:Malic acid X5:Citric acid	98.92	=−0.483–0.103 ×1 + 0.6660 X2 – 0.4301 ×3 + 0.485 X4 – 0.068 ×5 + 0.0287 X1*X4 + 0.02734 X2*X3 – 0.08320 X2*X4 – 0.02304 X2*X5 + 0.0479 X4*X5
Sn	X1:Oxalic acid X2:Malonic acid X3:Maleic acid X4:Malic acid X5:Citric acid	94.70	= 0.0083–0.00895 ×1 + 0.00912 X2 + 0.00667 X3 + 0.02020 X4- 0.00363 X5- 0.000985 X1*X2 + 0.001888 X1*X5 + 0.001120 X2*X3- 0.002018 X3*X5- 0.001089 X4*X5
Se	X1:Oxalic acid X2:Malonic acid X3:Succinic acid X4:Malic acid X5:Citric acid	95.36	= 1.650–0.1355 ×1 + 0.2883 X2- 0.1325 X3- 0.2532 ×4 + 0.1356 X5 + 0.02209 X1^2- 0.01859 X1*X4- 0.01991 X1*X5- 0.01724 X2*X4- 0.00890 X2*X5 + 0.01808 X3*X4 + 0.02928 X4*X5

The interaction of Oxalic acid, malonic acid, Malic acid, Lactic acid, Citric in phytochelatin of the Cd is explained at a rate of 67.89% (p < .05) (Table 2). Cd concentrations of the plants was estimated that an increase of 1 unit in the amount of Malonic acid and Malic acid would contribute to the chelation of 5.60 and 2.03 units of Cd in the plants. In a study, low concentrations of tartrate or citrate inhibited Cd desorption in soil, while high concentrations of citrate and tartrate promoted Cd desorption.^56^ Cr concentrations of the plants was estimated that an increase of 1 unit in the amount of Oxalic acid and Malic acid could contribute to the chelation of 0.09 and 0.2 units of Cr in the plants. Hg concentrations of the plants was estimated that an increase of 1 unit in the amount of Oxalic acid, Succinic acid, Citric acid, and Butyric acid could contribute to the chelation of 0.0009, 0.001, 0.01 and 0.0.003 units of Hg in the plants. Pb concentrations of the plants was estimated that an increase of 1 unit in the amount of Malonic acid could contribute to the chelation of 4.41 units of Pb in the plants. Ni concentrations of the plants was estimated that an increase of 1 unit in the amount of Malonic acid and Malic acid could contribute to the chelation of 0.7 and 0.5 units of Ni in the plants. Sn concentrations of the plants was estimated that an increase of 1 unit in the amount of Malonic acid, Maleic acid and Malic acid could contribute to the chelation of 0.009, 0.007 and 0.02 units of Sn in the plants. Se concentrations of the plants was estimated that an increase of 1 unit in the amount of Malonic acid and Citric acid could contribute to the chelation of 0.29 and 0.14 units of Se in the plants.

In a study on the *canola* plant, the changing levels of OAs showed important effects on all properties, including the accumulation of Pb in roots and stems.^57^ Cd accumulates in shoots, whereas Cr accumulates in the roots, shoots, leaves, and the dry weight of plants. The interaction with OAs changes the concentrations of the accumulation of Pb in roots and shoots, Cd in shoots, and Cr in roots, shoots, and leaves. It also showed its significance in the dry weight of the plant.^57^

Plants come up with strategies to counter the negative effects of HMs.^58^ The toxicity of HMs in a plant can result in excessive production of ROS, which causes the peroxidation of many vital cell components.^59^ For this reason, plants have an effective defense system consisting of enzymatic and non-enzymatic antioxidants^59^ such as SOD, POD, and CAT. They effectively oxidize superoxide radicals to hydrogen peroxide and can directly detoxify ROS while converting non-enzymatic antioxidants of low molecular weight, such as proline, ascorbic acid, and glutathione,^60^ to water and oxygen.^61–63^ Enzymatic and non-enzymatic antioxidant groups quench a wide variety of toxic oxygen derivatives to protect cells from oxidative stress. Plants accumulate various metabolic products, including AAs, under HM and abiotic stresses.^64^

There were differences in the correlation between HMs, such as Ni, Cr, Hg, Pb, Se, Sn, and Cd and antioxidants, proline, MDA, and hydrogen peroxide in plant samples from both the PA and NPA. Although a positive (p < .001) correlation was observed between Cd content and proline in NPA plants, no significant correlation was seen in PA plant samples (p > .05). The interaction between Pb and proline was completely in contrast with that between Cd and proline. Although this interaction showed a positive correlation at p < .001 in PA samples. In conjunction with the increased in Cd and Pb doses in *Eruca sativa L*., an increase in Proline content was observed.^65^ No significant correlation was found in the NPA samples (p > .05). On the other hand, the quantity of proline in NPA samples showed a negative correlation with the level of Cr, Ni, Hg, and Sn (p < .001). The PA samples (p < .005) showed a positive correlation with Cr, Ni, Hg, and Sn (p < .001). In a study, There is a correlation between Pro level and heavy metal accumulation, with Cd is being the strongest inducer of Pro content in cells. This association indicated that the accumulation of free Pro corresponded to the absorption of metals by *S. amateurs* cells.^66^ While a negative correlation for Se was observed in plant samples collected from the NPA, it showed a positive correlation at p < .001 in plants collected from the PA.

While redox-active (Ni, Fe, and Cu) HMs instantly catalyze the formation of ROS by Fenton reactions, they induce osmotic stress by reducing inactive (Cd, Pb, and Zn) metals and antioxidants.^67^ HM stresses trigger osmotic stress, oxidative stress, and denaturation of proteins in plants. The rate of ROS clearance and acceleration of the antioxidant system lead to cellular responses such as the activation of the formation of stress proteins.^68^ In general, plants in reaction to stress produce antioxidant enzymes (SOD, CAT, POD) to prevent the harmful effects of ROS and contribute to the stress response.^69,70^ Plants produce organ-specific antioxidant defense components depending on the type of stress and developmental stage to cope with oxidative stress.^71^ As a result of our study, antioxidant enzyme activity showed a positive correlation with Cd content in plant samples from the NPA for CAT, POD, and SOD at p < .001, while it showed a positive correlation at p < .05 in plant samples collected from the PA. It showed similar results for detoxification with proline and H_2_O_2_ in samples containing HMs Cr, Ni, Hg, and Sn. In this study, we found that the highest content of all antioxidant enzymes, except proline, was in the leaves. On the other hand, proline was highest in the stem region of the plant (S10). Proline content varied by type of heavy metals and among organs. These results can be a defense mechanism by Proline’s chelating capacity and its binding to metal ions. In a study of Cd applied to mung beans, Proline showed a negatively correlated with antioxidant enzymes. However, Proline content was not significant with MDA. It was also reported similar result by^72^ that their study conducted with *Spirulina platensis-S5*, an increase in both proline and MDA contents occurred with increasing metal ion concentration. Moreover, they reported a correlation between free radical formation and proline accumulation.^72^ In addationaly, all antioxidant enzymes and MDA content were also found to have a significant positive correlation.^73^ In our studied showed an increase in proline and MDA levels with an increase in the concentration of metallic ions. The fact that this increase was high in the stem portion of the plant was indicative of a correlation between free radical formation and proline accumulation (Figure 4). The activities of CAT, SOD, and POD showed higher total activity in the leaves.

H_2_O_2_ has a long lifespan and is highly permeable.^74^ It is through the membrane and can quickly diffuse from cell to cell within plants. Therefore, it is reactive, and is easily controlled by antioxidant enzymes as it is quickly produced.^74^ Based on these results, it is possible to conclude that the increase in H_2_O_2_ production in plants with a high heavy metal content may be linked to the high antioxidant activity observed (Figure 4). H_2_O_2_ provides all important criteria for intercellular signals to a high degree. Therefore, it should be considered as an Oxidative signaling molecule.^74^ Moreover, H_2_O_2_ and MDA not only act as signaling molecules under the stress of heavy metals but also play a major role in many physiological events, such as hypersensitive responses and stomatal conductance.^74^

In plants, HM stress and detoxification of ROS species are associated with proline accumulation. In this way, it protects the membrane integrity of the plant cell and regulates the osmoregulation of the cell, thereby protecting plants against oxidative damage.^63,75–77^ In this study, the amount of H_2_O_2_ in the plant samples showed a parallel correlation with proline.

## Conclusion

The exogenous application of different organic or inorganic compounds and their curative effects on HM-induced toxicity in plants are promising studies to clean up polluted land and improve crops. Therefore, it is important to understand the tolerance mechanisms for plant species in the natural environment against such stresses. Mechanisms that strengthen the defense mechanism in the natural environment should be supplied to the plant artificially. Because it’s a need-to-know to try to understand how exposure to HM contributes to the development of crop plants.

This research work aimed at identifying the different approaches to transportation and internal chelation of metals by AAs and OAs in plants. A multiple linear regression model was used to identify the AAs and OAs that jointly affect the chelation and stabilization of HMs in plants in response to metal toxicity and their force of action.

Related to our hypothesis, we can conclude the effect of heavy metals in the plant on phytochelation ability on the increase from one to five (pmol μL^−1^) of various types of amino acids as follow; Gly and Leu (%19.41, Cd), Trp and Ile (11.32%, Pb), Asp, Ser, and Leu (42.57%, Cr), Trp and Glu (25%, Ni). The effect of heavy metals in the plant on phytochelation ability on the increase from 0.1 to 0.5 (ng/microgram) of various types of Organic acids as follow; Malonic acid and Malic (19.16%, Cd), Malonic acid (10.37%, Pb), Malonic acid and Malic acid (445% Ni). Regression analysis consists of determining the relationship between two or more variables that have a cause and effect relationship. Used this relationship, it is made in order to make an estimation or a prediction on this topic. It is also possible to find a cause and effect relationship with many events in the wild. This estimation can show another aproach to improve heavy metal uptaking by plants and remediation of soils. Therefore, this research needs to in vitro experimental results.

Ser alone acted in Hg phytochelatin at the rate of 92.17%. This regression effect demonstrated that Ser may be an important AA in Hg chelation. For this reason, studies on the role of serine in Hg uptake and chelation should be undertaken

With the increase in the concentration of metal ions, the levels of proline and MDA increased significantly in the stem part of the plant. This increase points to a correlation between free radical formation and proline concentration. The CAT, SOD and POD activities showed a higher total activity in the leaves. It can be concluded that increased production of H_2_O_2_ in plants leaves with a high heavy metal content can be associated with high antioxidant activity. In addationally, Proline content varied by type of heavy metals and among organs. Therefore, these results can be a defense mechanism by Proline’s chelating capacity and related its binding to metal ions.

## Supplementary Material

Supplemental Material
