# Supplementary material for: Combined interactions of amino acids and organic acids in heavy metal binding in plants
Source: Plant Signal Behav. 2022 May 2;18(1):2064072. doi: 10.1080/15592324.2022.2064072 (PMC9980588; doi:10.1080/15592324.2022.2064072)
Supplement: Supplemental Material [file KPSB_A_2064072_SM2586.docx]

**S1**  TF and BAF values for four different plants from PA.

| **Species** | **TF** | | | | | | |
| --- | --- | --- | --- | --- | --- | --- | --- |
|  | **Cd** | **Cr** | **Hg** | **Pb** | **Ni** | **Sn** | **Se** |
| **SP** | 1.30 | 4.98 | 21.21 | 1.86 | 34.27 | 6.03 | 1.63 |
| **EA** | 1.32 | 5.17 | 15.63 | 1.89 | 35.40 | 6.66 | 1.49 |
| **TA** | 1.37 | 6.54 | 13.97 | 1.74 | 30.96 | 4.88 | 1.69 |
| **SA** | 1.46 | 8.07 | 13.26 | 1.90 | 13.26 | 5.79 | 1.90 |
|  | **BAF** | | | | | | |
|  | **Cd** | **Cr** | **Hg** | **Pb** | **Ni** | **Sn** | **Se** |
| **SP** | 12.33 | 0.54 | 0.19 | 6.27 | 25.72 | 1.02 | 1.77 |
| **EA** | 15.28 | 0.71 | 0.21 | 7.41 | 29.29 | 1.15 | 2.62 |
| **TA** | 21.12 | 0.97 | 0.23 | 6.98 | 27.82 | 1.10 | 1.58 |
| **SA** | 28.69 | 1.34 | 0.16 | 6.42 | 23.36 | 1.01 | 1.65 |

**S2**  Analysis results of soil samples taken from plant root zones

| Area | Plant species | pH | EC  µhos /cm | Calcif  % | OM  % |
| --- | --- | --- | --- | --- | --- |
| PA | ***SP*** | 6.77 | 104.70 | 0.40 | 2.17 |
| NPA | ***SP*** | 7.12 | 62.96 | 0.62 | 2.05 |
| PA | ***EA*** | 6.77 | 89.60 | 0.50 | 0.48 |
| NPA | ***EA*** | 7.27 | 64.47 | 0.86 | 2.68 |
| PA | ***TA*** | 7.21 | 86.96 | 0.83 | 0.67 |
| NPA | ***TA*** | 7.13 | 69.10 | 0.84 | 2.28 |
| PA | ***SA*** | 7.27 | 89.69 | 0.88 | 0.64 |
| NPA | ***SA*** | 6.98 | 72.70 | 0.91 | 2.00 |

**S3** Results of nutrient content analysis for soil samples from root areas.

| Area | Plant species | ttl N  % | P  ppm | K  cmol/kg | Ca  ppm | Mg  cmol/kg | Na  cmol/kg | B  ppm | Cl  ppm | Cu  ppm | Fe  ppm | Zn  ppm | Mn  ppm |
| --- | --- | --- | --- | --- | --- | --- | --- | --- | --- | --- | --- | --- | --- |
| PA | ***SP*** | 0.06 | 45.65 | 3.02 | 16.78 | 9.22 | 2.58 | 0.03 | 59.32 | 0.15 | 0.63 | 0.11 | 0.08 |
| NPA | ***SP*** | 0.06 | 34.13 | 2.85 | 15.85 | 15.86 | 1.58 | 0.02 | 1.01 | 0.56 | 1.31 | 0.13 | 0.11 |
| PA | ***EA*** | 0.01 | 23.20 | 0.66 | 3.69 | 7.78 | 2.34 | 0.02 | 34.24 | 0.16 | 0.47 | 0.13 | 0.07 |
| NPA | ***EA*** | 0.08 | 50.84 | 3.73 | 20.72 | 12.87 | 1.13 | 0.17 | 0.58 | 0.52 | 1.42 | 0.20 | 0.19 |
| PA | ***TA*** | 0.02 | 31.70 | 0.94 | 5.20 | 14.01 | 2.47 | 0.05 | 33.18 | 0.39 | 1.23 | 0.14 | 0.14 |
| NPA | ***TA*** | 0.07 | 49.45 | 3.16 | 17.58 | 12.80 | 1.30 | 0.15 | 0.56 | 0.44 | 1.28 | 0.21 | 0.28 |
| PA | ***SA*** | 0.02 | 30.43 | 0.88 | 4.92 | 15.73 | 2.73 | 0.05 | 30.53 | 0.39 | 1.32 | 0.16 | 0.14 |
| NPA | ***SA*** | 0.06 | 64.33 | 2.78 | 15.43 | 12.22 | 7.17 | 0.17 | 0.52 | 0.48 | 1.48 | 0.21 | 0.33 |

**S4** Results of analysis of heavy metal content of soil samples obtained from plant roots.

| Area | Plant species | Cd | Cr | Hg | Pb | Ni | Sn | Se |
| --- | --- | --- | --- | --- | --- | --- | --- | --- |
|  |  | ppm | | | | | | |
| PA | ***SP*** | 5.09 | 7.02 | 3.65 | 26.63 | 1.64 | 1.13 | 6.22 |
| NPA | ***SP*** | 0.04 | 0.06 | 0.02 | 0.13 | 0.01 | 0.01 | 0.04 |
| PA | ***EA*** | 5.09 | 10.22 | 3.99 | 27.91 | 1.78 | 0.87 | 5.08 |
| NPA | ***EA*** | 0.04 | 0.09 | 0.03 | 0.14 | 0.01 | 0.01 | 0.03 |
| PA | ***TA*** | 5.42 | 7.20 | 3.38 | 27.30 | 1.65 | 0.89 | 9.86 |
| NPA | ***TA*** | 0.04 | 0.06 | 0.02 | 0.14 | 0.01 | 0.01 | 0.07 |
| PA | ***SA*** | 5.46 | 7.74 | 4.75 | 31.40 | 2.16 | 0.96 | 9.85 |
| NPA | ***SA*** | 0.04 | 0.07 | 0.03 | 0.16 | 0.02 | 0.01 | 0.06 |

**S5** The mean nutrient content of plant samples collected from NPA and PA.

|  |  | |  | N | P | K | Ca | Mg | Zn | Fe | Cu | Cl | Na | B |
| --- | --- | --- | --- | --- | --- | --- | --- | --- | --- | --- | --- | --- | --- | --- |
| Area | | **Plants** | **Organ** | **%** |  |  |  |  | ppm |  |  |  |  |  |
| NPA | ***EA*** | | **root** | 1.79c | 908.07c | 2823.98d | 1722.43d | 766.31c | 9.65d | 34.54b | 16.54b | 0.32f | 484.23d | 13.38c |
|  |  |  | **stem** | 2.67a | 3015.46a | 22836.90a | 14279.48a | 890.18b | 98.36a | 11.12d | 15.17bc | 5.35c | **2834.60a** | **65.25a** |
|  |  |  | **leaf** | 2.17b | 2027.02b | 21054.90c | 5154.27b | 1616.93a | 16.87c | 97.78a | 26.21a | 0.74e | 593.19cd | 7.84cd |
| PA |  |  | **root** | .66e | 544.75d | 2176.06d | 1302.48d | 672.27c | 5.06d | 22.11c | 12.18c | 10.11b | 532.78cd | 6.59d |
|  |  |  | **stem** | .99d | 698.02cd | 6165.96d | 3855.46c | 240.35d | 26.56b | 3.00d | 4.09d | 2.02d | 765.34b | 30.21b |
|  |  |  | **leaf** | 1.44c | 2214.33b | 23454.50a | 5138.91b | 1663.41a | 15.67c | 91.57a | 25.78a | **21.92a** | 629.63c | 8.85cd |
| NPA | ***TA*** | | **root** | 2.42c | 859.56b | 3202.32cd | 1677.34c | 806.28c | 9.52d | 34.84b | 15.71b | 0.44c | 510.50c | 13.58b |
|  |  |  | **stem** | 3.61a | 3195.64a | 34796.13a | 22788.39a | 1239.30b | 156.77a | 14.02b | 19.44b | 6.29bc | **3389.87a** | **29.83a** |
|  |  |  | **leaf** | 2.76b | 2784.23a | 24976.34b | 6428.92b | 1906.30a | 23.15c | 117.21a | 33.51a | 0.92c | 563.81c | 11.90b |
| PA |  |  | **root** | 0.90f | 707.05b | 2501.08d | 1544.67c | 765.86c | 7.34d | 26.00b | 13.40b | 13.26b | 604.23c | 8.69b |
|  |  |  | **stem** | 1.34e | 739.73b | 9394.95c | 6152.86b | 334.61d | 42.33b | 3.79b | 5.25c | 2.53c | 915.26b | 13.81b |
|  |  |  | **leaf** | 1.67d | 2787.73a | 25670.87b | 6094.45b | 1894.97a | 22.74c | 107.67a | 28.38a | **25.77a** | 714.07bc | 11.67b |
| NPA | ***SA*** | | **root** | 2.09c | 885.62d | 3256.89d | 1709.01e | 797.86d | 9.91d | 36.57c | 16.43b | 0.50e | 452.12d | 15.71c |
|  |  |  | **stem** | 3.12a | 4198.72a | 26636.96b | 16965.45a | 1067.34c | 119.01a | 12.97d | 17.69b | 7.58c | **3306.28a** | **76.10a** |
|  |  |  | **leaf** | 2.71b | 3307.73b | 34103.00a | 6751.95b | 2058.71a | 28.30b | 141.08a | 33.41a | 1.07e | 655.44c | 16.63c |
| PA |  |  | **root** | 0.77f | 687.51d | 2401.15d | 1469.45e | 730.45d | 7.20d | 31.32c | 15.01b | 14.24b | 442.41d | 10.30d |
|  |  |  | **stem** | 1.15e | 838.59d | 7191.98c | 4580.67d | 288.18e | 32.13b | 3.50e | 4.78c | 2.94d | 892.70a | 35.23b |
|  |  |  | **leaf** | 1.68d | 2670.19c | 26174.65b | 5797.68c | 1807.36b | 22.28c | 129.73b | 31.79a | **31.06a** | 522.83d | 13.84cd |
| NPA | ***SP*** | | **root** | 1.68c | 657.30d | 2407.27e | 1547,23d | 725.37c | 8.46c | 31.58 c | 14.47b | 0.47e | 443.43c | 12.34d |
|  |  |  | **stem** | 2.52b | 4709.58a | 22321.01c | 15286,89a | 1015.17b | 95.80a | 17.50d | 16.21b | 7.63c | **2791.58a** | **82.23a** |
|  |  |  | **leaf** | 2.73a | 3478.53b | 34500.50a | 6644,29b | 1968.63a | 30.86b | 145.07a | 33.26a | 1.12de | 578.81bc | 19.45c |
| PA |  |  | **root** | 0.62e | 608.95d | 2266.66e | 1578,62d | 807.14c | 7.41c | 31.56c | 14.96b | 14.38b | 445.37c | 10.36d |
|  |  |  | **stem** | 0.93d | 844.50d | 6026.67d | 4127,46c | 274.10d | 25.87b | 2.21e | 4.38c | 3.06d | 753.73b | 69.09b |
|  |  |  | **leaf** | 1.64c | 2800.93c | 26198.54b | 6228,40b | 1997.10a | 22.93b | 130.69b | 31.68a | **31.29a** | 526.32c | 13.91d |

**S6** Means of heavy metal content in plant organs.

| **area** | **plants** | **organ** | **Cd** | **Cr** | **Hg** | **Pb** | **Ni** | **Sn** | **Se** |
| --- | --- | --- | --- | --- | --- | --- | --- | --- | --- |
|  |  |  | **ppm** | | | | | | |
| **NPA** | ***EA*** | **root** | 0.33d | 0.13c | 0.02c | 0.24d | 0.15c | 0.03c | 0.00d |
|  |  | **stem** | 0.46d | 0.04e | 0.00d | 0.45d | 0.07d | 0.01d | 0.00d |
|  |  | **leaf** | 0.70d | 0.05d | 0.00d | 0.61d | 0.10d | 0.02d | 0.00d |
| **PA** |  | **root** | 4.86c | 0.10cd | 0.01d | 10.40c | 0.16d | 0.03c | 0.72c |
|  |  | **stem** | 6.33b | 0.52b | 0.10b | 19.30b | 5.48b | 0.15b | 1.17b |
|  |  | **leaf** | 9.71a | 0.64a | 0.12a | 25.96a | 8.40a | 0.21a | 1.79a |
| **NPA** | ***TA*** | **root** | 0.39c | 0.18c | 0.02c | 0.31d | 1.28c | 0.03c | 0.00c |
|  |  | **stem** | 0.59c | 0.05c | 0.00c | 0.57d | 0.08d | 0.01c | 0.00c |
|  |  | **leaf** | 0.91c | 0.06c | 0.00c | 0.76d | 0.12d | 0.01c | 0.00c |
| **PA** |  | **root** | 5.96b | 0.13c | 0.01c | 12.10c | 0.18d | 0.03c | 1.14b |
|  |  | **stem** | 7.87b | 0.69b | 0.11b | 22.89b | 6.24b | 0.17b | 1.69ab |
|  |  | **leaf** | 12.07a | 0.84a | 0.13a | 30.78a | 9.57a | 0.23a | 2.60a |
| **NPA** | ***SA*** | **root** | 0.39c | 0.25b | 0.02c | 0.31d | 1.30c | 0.02c | 0.00d |
|  |  | **stem** | 0.58c | 0.06b | 0.00d | 0.60d | 0.08d | 0.01d | 0.00d |
|  |  | **leaf** | 0.90c | 0.07b | 0.01d | 0.80d | 0.13d | 0.02cd | 0.00d |
| **PA** |  | **root** | 8.53b | 0.15b | 0.01d | 12.48c | 0.19d | 0.03c | 0.98c |
|  |  | **stem** | 11.68ab | 0.98a | 0.12b | 21.78b | 5.95b | 0.13b | 1.66b |
|  |  | **leaf** | 17.93a | 1.20a | 0.13a | 29.28a | 9.13a | 0.17a | 2.55a |
| **NPA** | ***SP*** | **root** | 0.46d | 0.23c | 0.01c | 1.22d | 0.95c | 0.02c | 0.07c |
|  |  | **stem** | 0.63d | 0.07c | 0.01c | 0.60d | 0.08d | 0.01c | 0.00c |
|  |  | **leaf** | 0.96d | 0.08c | 0.01c | 0.80d | 0.12d | 0.01c | 0.00c |
| **PA** |  | **root** | 11.09c | 0.18c | 0.01c | 12.32c | 0.20d | 0.02c | 1.09c |
|  |  | **stem** | 16.24b | 1.47b | 0.12b | 23.40b | 6.57b | 0.13b | 1.71b |
|  |  | **leaf** | 24.92a | 1.80a | 0.13a | 31.46a | 10.09a | 0.17a | 2.62a |

**SI 7** Means of amino acid contents of plant samples.

| area | plants | organ | Asp | Glu | Asn | Ser | Gln | His | Giy | Thr | Arg | Ala |
| --- | --- | --- | --- | --- | --- | --- | --- | --- | --- | --- | --- | --- |
|  |  |  | **pmol μL−1** | | | | | | | | | |
| NPA | EA | **root** | 19.38c | 23.00c | 9.78e | 2.38c | 12.97c | 28.31c | 4.51d | 1.12d | 6.96c | 1.01c |
|  |  | **stem** | 186.69b | 114.60b | 217.01d | 189.43b | 112.90b | 138.78ab | 115.68c | 98.85c | 156.36b | 70.96bc |
|  |  | **leaf** | 349.53a | 184.35a | 315.75c | 392.82a | 149.61ab | 144.85ab | 194.56b | 195.30b | 341.94a | 199.54a |
| PA |  | **root** | 37.48c | 37.81c | 20.32e | 6.16c | 22.67c | 43.33c | 10.83d | 3.22d | 14.64c | 2.31c |
|  |  | **stem** | 361.03a | 188.43a | 450.91a | 491.11a | 197.31a | 212.43a | 277.99a | 283.26a | 328.68a | 162.19ab |
|  |  | **leaf** | 350.52a | 178.41a | 364.06b | 410.39a | 141.50ab | 122.73b | 173.20b | 231.41ab | 287.09a | 99.48abc |
| NPA | TA | **root** | 21.09d | 28.96c | 7.55c | 2.33d | 12.85c | 26.67c | 4.04d | 0.80c | 8.33d | 2.73b |
|  |  | **stem** | 203.18c | 144.31b | 167.59b | 186.00c | 111.89b | 130.75b | 103.83c | 70.50b | 187.10c | 191.91b |
|  |  | **leaf** | 420.99a | 146.73b | 337.68a | 350.28b | 134.94b | 143.7ab | 207.05ab | 207.27a | 254.85bc | 117.31b |
| PA |  | **root** | 40.79d | 47.61c | 15.69c | 6.05d | 22.47c | 40.82c | 9.72d | 2.29c | 17.52d | 6.24b |
|  |  | **stem** | 392.92b | 237.28a | 348.21a | 482.21a | 195.54a | 200.1a | 249.52a | 202.00a | 393.32a | 438.63a |
|  |  | **leaf** | 378.70b | 182.21ab | 316.12a | 395.94b | 146.73b | 131.92b | 187.35b | 209.90a | 303.35ab | 90.16b |
| NPA | SA | **root** | 18.81c | 24.39c | 9.04c | 1.75d | 10.59c | 24.90b | 3.48c | 0.78c | 8.45c | 2.02c |
|  |  | **stem** | 181.16b | 121.56b | 200.53b | 139.65c | 92.14b | 122.08a | 89.31b | 68.30b | 189.68b | 141.74bc |
|  |  | **leaf** | 353.10a | 212.91a | 366.46a | 441.88a | 180.09a | 190.61a | 231.30a | 202.74a | 388.10a | 390.42a |
| PA |  | **root** | 36.37c | 40.11c | 18.77c | 4.54d | 18.50c | 38.12b | 8.36c | 2.22c | 17.76c | 4.61c |
|  |  | **stem** | 350.33a | 199.87a | 416.66a | 362.05b | 161.03a | 186.87a | 214.63a | 195.73a | 398.74a | 323.96ab |
|  |  | **leaf** | 379.29a | 170.49ab | 364.77a | 390.09b | 152.76a | 150.65a | 201.59a | 227.79a | 276.34ab | 108.18c |
| NPA | SP | **root** | 15.58d | 23.75c | 7.15c | 1.78d | 9.19d | 20.29c | 2.67d | 0.69c | 7.22c | 1.78a |
|  |  | **stem** | 150.10c | 118.35b | 158.71b | 142.19c | 79.97c | 99.47b | 68.51c | 60.88b | 162.13b | 125.17 |
|  |  | **leaf** | 455.62a | 207.87a | 375.75a | 402.71b | 140.88b | 145.82a | 221.07a | 187.00a | 397.58a | 317.96a |
| PA |  | **root** | 30.14d | 39.05c | 14.86c | 4.63d | 16.06d | 31.06c | 6.41d | 1.98c | 15.18c | 4.07a |
|  |  | **stem** | 290.26b | 194.59a | 329.77a | 368.63b | 139.75b | 152.25a | 164.65b | 174.47b | 340.82a | 286.09 |
|  |  | **leaf** | 395.34a | 219.92a | 365.16a | 488.22a | 170.49a | 148.13a | 203.02a | 255.73a | 327.12a | 116.61a |
| area | **plants** | **organ** | **Tyr** | **Cys** | **Val** | **Met** | **Trp** | **Phe** | **Ile** | **Leu** | **Lys** | **Sarcosine** |
|  |  |  | **pmol μL−1** | | | | | | | | | |
| NPA | EA | **root** | 24.15d | 15.33c | 21.68c | 2.86c | 3.74c | 63.25d | 7.68b | 2.49b | 1.47b | 11.86c |
|  |  | **stem** | 232.56c | 125.53b | 124.13bc | 69.39b | 252.35b | 509.54c | 50.87ab | 66.71b | 108.72ab | 112.85b |
|  |  | **leaf** | 354.24b | 158.86b | 223.83ab | 179.84a | 277.93b | 578.24bc | 231.59a | 232.66a | 371.85a | 256.12a |
| PA |  | **root** | 60.45d | 27.74c | 51.78c | 8.16c | 7.65c | 110.77d | 24.54b | 8.67b | 3.60b | 28.98c |
|  |  | **stem** | 582.21a | 227.16a | 296.43a | 198.09a | 515.91a | 892.43a | 162.51ab | 232.65a | 265.76ab | 275.84a |
|  |  | **leaf** | 369.56b | 134.09b | 216.05ab | 151.70a | 302.03b | 629.41b | 111.31ab | 144.49ab | 180.91ab | 221.86a |
| NPA | TA | **root** | 18.98d | 16.56c | 20.35d | 3.86c | 2.37c | 46.37d | 16.55b | 3.26c | 3.45b | 15.36d |
|  |  | **stem** | 182.85c | 135.57b | 116.51c | 93.60b | 160.08b | 373.59c | 109.60b | 87.54bc | 254.38b | 146.20bc |
|  |  | **leaf** | 441.96a | 141.26b | 199.52b | 159.98b | 364.02a | 676.50a | 112.11b | 132.62b | 186.20b | 226.07b |
| PA |  | **root** | 47.52d | 29.96c | 48.60cd | 11.01c | 4.85c | 81.22d | 52.88b | 11.38c | 8.43b | 37.55cd |
|  |  | **stem** | 457.74a | 245.33a | 278.23a | 267.18a | 327.27a | 654.32ab | 350.13a | 305.30a | 621.80a | 357.36a |
|  |  | **leaf** | 343.84b | 136.91a | 213.55ab | 152.33b | 309.17a | 593.22c | 106.99b | 140.82b | 201.18b | 213.77b |
| NPA | SA | **root** | 16.85c | 12.13d | 18.62c | 3.13b | 2.07c | 47.45c | 16.17c | 2.98b | 2.80c | 13.99c |
|  |  | **stem** | 162.31b | 99.33c | 106.58b | 75.83b | 139.89b | 382.24b | 107.03b | 79.96b | 206.78ab | 133.15bc |
|  |  | **leaf** | 414.80a | 211.20a | 262.73a | 242.48a | 291.86a | 632.34a | 343.26a | 310.92a | 560.08a | 359.54a |
| PA |  | **root** | 42.19 | 21.95d | 44.46c | 8.92b | 4.24c | 83.10c | 51.64c | 10.40b | 6.85c | 34.20c |
|  |  | **stem** | 406.34a | 179.76ab | 254.52a | 216.46a | 285.99a | 669.46a | 341.93a | 278.87a | 505.44ab | 325.47a |
|  |  | **leaf** | 410.46a | 142.62b | 221.10a | 171.30a | 349.47a | 657.49a | 117.14b | 140.90b | 192.90bc | 246.74ab |
| NPA | SP | **root** | 13.31c | 11.32c | 17.08c | 2.77c | 1.57d | 35.58d | 13.32c | 2.72c | 2.43b | 12.09c |
|  |  | **stem** | 128.24c | 92.73b | 97.75b | 67.13b | 105.79c | 286.65c | 88.17abc | 73.02bc | 179.13ab | 115.03b |
|  |  | **leaf** | 507.84a | 193.66a | 216.81a | 190.27a | 344.69a | 773.39a | 238.09ab | 209.23a | 448.51a | 242.12 |
| PA |  | **root** | 33.33c | 20.49c | 40.78c | 7.90c | 3.21d | 62.32d | 42.54bc | 9.50c | 5.94b | 29.54c |
|  |  | **stem** | 321.04b | 167.80a | 233.44a | 191.62a | 216.29b | 502.05b | 281.68a | 254.69a | 437.86a | 281.16a |
|  |  | **leaf** | 450.64a | 170.88a | 254.07a | 178.62a | 369.80a | 712.49a | 126.70abc | 160.92ab | 227.76ab | 239.40a |

**SI 8** Mean organic acid content of plants.

| **area** | **plants** | **organ** | **Oxalic acid** | **Propionic acid** | **Tartaric acid** | **Bütyric acid** | **Malonic acid** | **Malic acid** | **Lactic acid** | **Citric acid** | **Maleic acid** | **Fumaric acid** | **Succinic acid** |
| --- | --- | --- | --- | --- | --- | --- | --- | --- | --- | --- | --- | --- | --- |
|  |  |  | **ng/mikrogram** | | | | | | | | | | |
| **NPA** | **EA** | **root** | 0.94d | 1.49c | 1.53d | 0.40e | 0.26c | 1.25c | 1.33d | 0.22c | 0.03c | 0.19c | 1.25c |
|  |  | **stem** | 9.03c | 12.18b | 8.77c | 9.78d | 17.30b | 10.10b | 8.82cd | 5.85b | 2.57bc | 13.40b | 11.89b |
|  |  | **leaf** | **10.94c** | **14.19b** | **9.59c** | **13.73c** | **19.48b** | **9.71b** | 13.18bc | **18.48a** | 7.46a | 14.05b | 23.59a |
| **PA** |  | **root** | 2.35d | 2.69c | 3.66d | 1.15e | 0.52c | 2.19c | 4.25cd | 0.76c | 0.09c | 0.42c | 3.05c |
|  |  | **stem** | **22.59a** | **22.05a** | **20.95a** | **27.92a** | **35.37a** | **17.68a** | **28.17a** | **20.42a** | **6.28ab** | **29.73a** | **29.06a** |
|  |  | **leaf** | 19.45b | 21.80a | 16.05b | 19.14b | 22.22b | 14.13ab | 19.65ab | 17.71a | 8.41a | 17.37b | 25.44a |
| **NPA** | **TA** | **root** | 0.48d | 0.74e | 0.99c | 0.28d | 0.22c | 0.76d | 1.00b | 0.24d | 0.03c | 0.13c | 1.21c |
|  |  | **stem** | 4.62d | 6.09d | 5.69c | 6.74c | 14.58b | 6.09c | 6.61b | 6.38c | 2.31bc | 9.22b | 11.55b |
|  |  | **leaf** | 18.39b | 18.95b | 20.46a | 28.96a | 30.95a | 12.59ab | **23.58a** | 16.41b | 6.55b | 22.33a | 29.16a |
| **PA** |  | **root** | 1.20d | 1.35e | 2.37c | 0.79d | 0.44c | 1.32d | 3.19b | 0.83d | 0.08c | 0.29c | 2.97c |
|  |  | **stem** | 11.57c | 11.02c | 13.59b | 19.25b | 29.81a | 10.67b | 21.11a | 22.25a | 5.66bc | 20.45a | 28.23a |
|  |  | **leaf** | **23.18a** | **22.66a** | **16.05ab** | **26.90a** | **25.60a** | **14.58a** | **20.04a** | **16.77b** | **14.46a** | **22.78a** | **25.73a** |
| **NPA** | **SA** | **root** | 0.71d | 1.28d | 1.03c | 0.29c | 0.16c | 0.70d | 0.78c | 0.20c | 0.05c | 0.10c | 0.97c |
|  |  | **stem** | 6.86d | 10.45c | 5.89b | 7.14b | 10.99b | 5.61c | 5.17c | 5.33b | 3.61bc | 7.13b | 9.24b |
|  |  | **leaf** | 13.10c | 15.77bc | 13.76a | 23.26a | 25.88a | 9.25b | 17.6b | 18.7a | 5.67ab | 16.63a | 28.09a |
| **PA** |  | **root** | 1.78d | 2.31d | 2.46bc | 0.84c | 0.33c | 1.22d | 2.49c | 0.69c | 0.12c | 0.23c | 2.37bc |
|  |  | **stem** | **17.1b** | **18.92ab** | **14.07a** | **20.39a** | **22.47a** | **9.83b** | **16.5b** | **18.5a** | **8.82a** | **15.81a** | **22.58a** |
|  |  | **leaf** | **21.42a** | **23.34a** | **17.42a** | **21.01a** | **22.26a** | **15.09a** | **22.53a** | **19.61a** | **7.97a** | **19.71a** | **25.86a** |
| NPA | **SP** | **root** | 0.69c | 1.22e | 1.05b | 0.29d | 0.17d | 0.70d | 0.76f | 0.19e | 0.05c | 0.11c | 1.01d |
|  |  | **stem** | 6.62b | 9.98c | 6.03b | **7.04c** | 11.20c | 5.68c | 5.04d | 5.00d | 3.44b | 7.47b | 9.63c |
|  |  | **leaf** | 9.28b | 10.68c | 12.15a | 13.21b | 23.59ab | 12.65a | 18.42b | 19.96a | 6.81a | 19.85a | 27.98a |
| **PA** |  | **root** | 1.72c | 2.21d | 2.52b | 0.83d | 0.34d | 1.23d | 2.43e | 0.65e | 0.11c | 0.24c | 2.47d |
|  |  | **stem** | 16.56a | 18.06b | 14.41a | 20.10a | 22.90b | 9.95b | 16.10c | 17.45b | 8.42a | 16.57a | 23.55b |
|  |  | **leaf** | **16.96a** | **19.16a** | **16.23a** | **22.74a** | **27.99a** | 10.71b | 19.94a | 14.77c | 8.11a | 16.52a | 30.52a |

**SI 9** Means of plants antioxidant enzymes, proline, H2O2, MDA content.

| **area** | **plants** | **organ** | **SOD** | **CAT** | **POX** | **PROLİN** | **H2O2** | **MDA** |
| --- | --- | --- | --- | --- | --- | --- | --- | --- |
| **NPA** | ***Echium angustifolium*** | **root** | 4.78e | 8.25e | 178.73e | 9.94d | 0.56e | 0.12e |
|  |  | **stem** | 402.59d | 169.31d | 11155.48d | 90.13c | 12.82d | 7.77d |
|  |  | **leaf** | 1828.89a | 470.28a | 27470.00a | 154.73b | 36.90a | 19.21a |
| **PA** |  | **root** | 15.26e | 16.86e | 313.04e | 22.05d | 1.41e | 0.22e |
|  |  | **stem** | 1286.11b | 346.14b | 19538.04b | 199.98a | 32.10b | 14.05b |
|  |  | **leaf** | 969.84c | 249.51c | 14345.07c | 141.40b | 18.93c | 10.09c |
| **NPA** | ***Typha angustifolia*** | **root** | 0.54e | 0.95e | 19.38e | 9.12d | 0.12e | 3.48d |
|  |  | **stem** | 45.50d | 19.56d | 1209.35d | 82.68c | 2.62d | 220.76b |
|  |  | **leaf** | 1078.96a | 271.76b | 15706.73b | 139.47b | 17.91b | 11.93c |
| **PA** |  | **root** | 1.72e | 1.95e | 33.94e | 20.23d | 0.29e | 6.30d |
|  |  | **stem** | 145.35c | 39.99c | 2118.09c | 183.45a | 6.57c | 399.49a |
|  |  | **leaf** | 1479.94a | 366.58a | 20997.36a | 141.31b | 23.32a | 15.47c |
| **NPA** | ***Sisymbrim austriacum*** | **root** | 0.46d | 0.90d | 16.58d | 8.34d | 0.11e | 2.97c |
|  |  | **stem** | 38.72cd | 18.55d | 1034.72cd | 75.61c | 2.46d | 188.48b |
|  |  | **leaf** | 1078.46b | 282.28b | 16184.95b | 183.30a | 21.71b | 11.92c |
| **PA** |  | **root** | 1.47d | 1.85d | 29.04d | 18.50d | 0.27e | 5.38c |
|  |  | **stem** | 123.70c | 37.92c | 1812.23c | 167.76ab | 6.16c | 341.08a |
|  |  | **leaf** | 1500.39a | 385.88a | 22422.73a | 147.48b | 30.30a | 15.68c |
| **NPA** | ***Setaria pumila*** | **root** | 0.46c | 0.93d | 16.74e | 8.11c | 0.11d | 3.05c |
|  |  | **stem** | 38.79c | 19.02d | 1044.70d | 73.56b | 2.48d | 193.42b |
|  |  | **leaf** | 1001.51b | 253.39b | 14756.78b | 151.22a | 11.28b | 13.90c |
| **PA** |  | **root** | 1.47c | 1.89e | 29.32e | 18.00c | 0.27d | 5.52c |
|  |  | **stem** | 123.92c | 38.88c | 1829.71c | 163.21a | 6.20c | 350.02a |
|  |  | **leaf** | 1449.91a | 395.41a | 21934.30a | 154.28a | 38.48a | 14.93c |

**SI 10** Antioxidant, proline, H_2_O_2_, MDA concentration distribution based on the heavy metal content in plant organs.
